# Population trend inferred from aural surveys for calling anurans in Korea

**DOI:** 10.7717/peerj.5568

**Published:** 2018-09-19

**Authors:** Amaël Borzée, Desiree Andersen, Yikweon Jang

**Affiliations:** 1 Laboratory of Behavioral Ecology and Evolution, School of Biological Sciences, Seoul National University, Seoul, South Korea; 2 Division of EcoScience, Ewha Women’s University, Seoul, South Korea; 3 Department of Life Sciences, Ewha Womans University, Seoul, Republic of Korea

**Keywords:** Aural survey, Population trend, Encroachment, Hylid, *Dryophytes suweonensis*, Republic of Korea

## Abstract

Amphibian populations fluctuate naturally in size and range and large datasets are required to establish trends in species dynamics. To determine population trends for the endangered Suweon Treefrog (*Dryophytes suweonensis*), we conducted aural surveys in 2015, 2016, and 2017 at each of 122 sites where the species was known to occur in the Republic of Korea. Despite being based on individual counts, the focus of this study was to establish population trends rather than population size estimates, and we found both environmental and landscape variables to be significant factors. Encroachment was also a key factor that influenced both the decreasing number of calling individuals and the negative population dynamics, represented here by the difference in the number of calling individuals between years. Generally, most sites displayed minimal differences in the number of calling males between years, although there was a large fluctuation in the number of individuals at some sites. Finally, when adjusted for the overall population size difference between years, we found the population size to be decreasing between 2015 and 2017, with a significant decrease in the number of calling individuals at specific sites. High rate of encroachment was the principal explanatory factor behind these marked negative peaks in population dynamics.

## Introduction

The current population decline in most organisms is so widespread that it has been termed the sixth mass extinction (Wake & Vredenburg, 2008). Among threatened groups, amphibians are in the worse position (Stuart et al., 2004; Pimm et al., 2014) due to a broad spectrum of causes (Beebee & Griffiths, 2005; Blaustein et al., 2011). However, it is difficult to ascertain the causes of population decline (Loehle & Weatherford, 2017), mainly due to a general low detectability of individuals, non-recurrent breeding, breeding at different sites over years, and highly variable populations sizes (Semlitsch et al., 1996; Taylor, Scott & Gibbons, 2006; Bevelhimer et al., 2008; McCain, Szewczyk & Bracy Knight, 2016). Besides, some taxa are easier than others to detect (Marsh & Trenham, 2008). For instance, it is difficult to detect non-calling cryptic species such as *Ambistoma cingulatum* (Bevelhimer et al., 2008), while species producing advertisement calls, such as *Hyla meridionalis*, can be detected more reliably (Petitot et al., 2014). Moreover, abundant species at a site have a higher probability of being detected than species with low population size (MacKenzie, 2006; Tanadini & Schmidt, 2011), and the experience of observers also has a direct impact on the detection probability (Fitzpatrick et al., 2009). Therefore, all amphibian population estimates may suffer from biases and inaccuracies that may not all be accounted in population dynamics models (MacKenzie, 2006; MacKenzie et al., 2009).

The use of long-term monitoring over large numbers of populations is important, as it enables conservationists to establish trends for population dynamics of focal species. For instance, Petrovan & Schmidt (2016) have used data provided by volunteers from Switzerland and the United Kingdom to establish population trends for the common European toad, *Bufo bufo*. Once a trend is established, it can be used to assess the status of a species through the International Union for Conservation of Nature (IUCN) red list (IUCN, 2017a) based on both distribution and abundance criteria (see Houlahan et al., 2000 or Cruickshank et al., 2016). The information can then be used to justify the implementation of conservation management plans (Mace, 2014). Therefore, it is of prime importance to establish population trends for the conservation of endangered species.

Besides natural variations in population and chorus sizes because of ecological variables and other co-occurring species (Houlahan et al., 2000; Phelps, Rand & Ryan, 2007; Llusia et al., 2013), anthropogenic factors also have a strong impact on population trends. For instance, a large number of man-made landscape features have a negative impact on the connectivity of populations (Forman & Alexander, 1998; Geneletti, 2004), a critical feature for the survival of species (Fahrig, 1997; Dodd Jr, 2010). In addition, urbanisation results in habitat loss, habitat fragmentation, and degradation of habitat quality, leading one third of amphibian species to be threatened (Hamer & McDonnell, 2008). The impacts of encroachment are insidious as the land is fractionally transformed and the impact of such slow-paced but constant destruction of the habitat is harder to pinpoint (Green, 1997; Curado, Hartel & Arntzen, 2011). Finally, and in relation to this study, the transformation of wetlands into rice-paddies (Park et al., 2005), and then their modernisation to increase crop yields also have negative effects on both population size (Moriyama, 1997; Fujioka & Lane, 1997; Kidera et al., 2018) and microhabitat use by frogs such as Hylids (Groffen, Borzée & Jang, 2018).

*Dryophytes suweonensis* (previously attributed to *Hyla*; Duellman, Marion & Hedges, 2016) is an endemic and threatened treefrog species from the Korean peninsula (Borzée, Yu & Jang, 2016a; Borzée et al., 2017a; IUCN, 2017b), with all known populations but one in the Republic of Korea, and the other one in the Democratic People’s Republic of Korea (Chun et al., 2012). The species is restricted to breeding in rice paddies (Borzée & Jang, 2015). It is sympatric with *D. japonicus*, a species ubiquitously present in wetlands, and with which it competes for calling space and microhabitat (Borzée et al., 2016b; Borzée, Kim & Jang, 2016c). The range of this species in the Republic of Korea has been clearly defined (Borzée et al., 2017a), and the number of sites where it occurs is decreasing because of encroachment. However, no population trend has been established for this species. Our aim was to evaluate how the abundance of calling male *D. suweonensis* in rice paddies changes with environmental variables, anthropogenic variables and the abundance and occurrence of other frog species. Because of difficulties and uncertainty in estimating population sizes for calling anurans, the focus of this study is a comparative analysis in population trends rather than population size estimates. In this study, we surveyed yearly between 2015 and 2017 all sites where *D. suweonensis* was known to occur and related population size fluctuations to environmental factors. We hypothesized that population sizes of *D. suweonensis* would not significantly fluctuate because hydrological, and environmental conditions surrounding rice paddies would remain relatively constant between years. This study highlights the importance of acoustic surveys in monitoring calling anuran populations and the methods followed here can be used to develop protocols for similar or different environments, where understanding the status and distribution of specific species is required.

## Material and Methods

The observations made in this study were based on non-invasive call monitoring. Laws of the Republic of Korea do not require research permits for such non-invasive surveys.

### Species and habitat description

Advertisement calls of *D. suweonensis* and *D. japonicus* are species specific (Jang et al., 2011; Park, Jeong & Jang, 2013) and are suitable for field surveys even to untrained ears (Roh, Borzée & Jang, 2014; Borzée et al., 2015; Borzée et al., 2017b). In calling Hylids, acoustic monitoring is used to estimate population sizes (Weir et al., 2005; Pellet, Helfer & Yannic, 2007; Dorcas et al., 2009; Petitot et al., 2014; Moreira, Moura & Maltchik, 2016), and the aural survey protocol followed here is accurate to estimate occurrence (Borzée et al., 2017a; Borzée et al., 2017b). However, population surveys of closely related calling Hylid species have demonstrated that most individuals are detected within two minutes of survey (Petitot et al., 2014; Corn et al., 2011). By walking along a transect line, and because the detection range of the species is roughly 250 m (Borzée et al., 2017b), we estimated our survey protocol to be also adequate to assess the number of calling males at each site.

Surveys were facilitated by the modern setting of rice fields. Agricultural developments during the last decades have led to a specific geometric grouping of rice paddies, here referred to as rice-paddy complexes. A rice-paddy complex is characterized by a central ditch running mostly straight through the complex for irrigation purposes. Along this central ditch, a cemented lane usually runs along the longest and straightest line available, typically following the centre of the valley.

### Survey protocol

Selection of populations surveyed in this study was based on country-wide field surveys that determined the occurrence of *D. suweonensis* at 122 sites in 2014 (Borzée et al., 2017a) and on data presented by Roh, Borzée & Jang (2014). These surveys were conducted at 116 of 122 sites where the species occurred during the breeding seasons of 2015, 2016, and 2017. This time span corresponds to about one half to one third of the life span of the species, estimated from *D. cinereus* (six years; Nichols, 2008), *D. versicolor* (seven years; Mueller, 2006a), *D. chrysoscelis* (seven years; Mueller, 2006b) and *D. japonicus* (nine years; AmphibiaWeb, 2017).

Six of the 122 sites where the species is known to occur were not included in this study, as access to these sites requires special permits that were not unavailable for all of the three years of this study. During each survey, we counted the number of calling males as a proxy for population size. These surveys were conducted following a protocol robust to variations in occurrence between replicates, although untested for population size estimates (Borzée et al., 2017b). To achieve maximum detectability of calling males, surveys were performed after rice plantation (Borzée & Jang, 2017) and at the beginning of diel calling activity (Roh, Borzée & Jang, 2014; Borzée, Kim & Jang, 2016c). The protocol followed the one described by Borzée et al. (2017a) as follows: “after arrival at a survey site, five minutes were spent waiting quietly. For each site, aural monitoring was conducted along a single transect along the centre of the rice-paddy complex. A surveyor walked briskly at a maximum speed of *circa* 80 m/min along the transect, counting the number of *D. suweonensis* calling at the rice-paddy complex. The detection range for advertisement calls of the species was empirically measured prior to surveys, resulting in a 250 ± 45 m detection range (*n* = 20). All rice paddies within the rice-paddy complexes surveyed were typically within the detection range”.

At the end of each survey, we also recorded date, time, air temperature (°C), air relative humidity (%), air pressure (hPa), light intensity (lx), moon phase (%), water temperature (°C), water pH, and water conductivity (µS). We also estimated scaled variables following the methodology set by Johnson & Batie (2001): wind speed (scale 1–4), cloud cover (scale 1–5), precipitation (scale 1–4), and night time cover (scale 1–5). Finally, we collected data for calling indices (CI) of *D. japonicus*, *Pelophylax nigromaculatus*, *P. chosenicus* (also producing species specific calls, Yoo & Jang, 2012), and the presence-absence of *Lithobates catesbeianus* at the site. The calling index was defined according to Mossman et al. (1998): 0, no frog calling; 1, non-overlapping and countable calling individuals; 2, overlapping and countable counting individuals; and 3, overlapping and non-countable individuals. Despite acknowledged the weaknesses of the CI methodology (Corn et al., 2011), it is not assumed to have any impact of downstream analyses. Finally, this study is not subject to interobserver variations (Shirose et al., 1997; Pierce & Gutzwiller, 2007) as all surveys were conducted by the same person.

Finally, for each year, we recorded whether encroachment occurred in each rice-paddy complex. Because of the widespread occurrence of encroachment, the modification of up to four contiguous rice paddies within a rice-paddy complex would have led to all rice-paddy complexes to be encoded positive for encroachment. As a result, a threshold of at least six contiguous modified rice paddies within a rice-paddy complex was set for a rice-paddy complex to be defined as under encroachment pressure. Encroachment was binary encoded for the year when it was detected and for subsequent years, as effects are not reversed even if the extent of the encroachment does not expend. Common examples of encroachment were road construction and building construction, but also included development of power plants, golf fields, and quarries, in decreasing frequency. In this analysis, we did not include the conversion of rice paddies to dry agriculture and greenhouses as these occurred at all sites where this species was present. Such conversion would have to be quantified over the years to be incorporated in the analysis.

### Statistical analysis

All variables were first verified for correlations to avoid collinearity in subsequent analyses. Because water temperature was correlated with air temperature (*n* = 363; Pearson Correlations; *r* = 0.83, *p* < 0.001), this variable was not used for subsequent analyses. No other abiotic variables were significantly correlated with each other.

#### Relationship between number of individuals and environmental variables

We first analysed the effect of environmental and landscape variables listed above, focusing on the effect of encroachment on the calling activity of the species. This analysis was biased as the species was known to be present at all sites surveyed, although the number of calling individuals was different at each site. For this analysis, we selected a Generalised Linear Mixed Model (GLMM) under a Poisson distribution (Bolker et al., 2009). The number of calling *D. suweonensis* was the dependent variable while predictor variables were divided in three groups: (1) random effect (site); (2) factors (date, time of day); and (3) covariates (wind speed, cloud cover, rain intensity, light intensity, air pressure, night cover, moon percentage, water pH, water conductivity, air temperature, CI of *D. japonicus*, *P. nigromaculatus*, *P. chosenicus*, occurrence of *L. catesbeianus*, and encroachment). All variables were run under a main effect model, with a model-based covariance matrix calculated though maximum likelihood estimates.

The model was run under these variables as no assumption was violated. Visual inspection of scatterplots and partial regression plots demonstrated a linear relationship between the dependent variable and each independent variable, or between the dependent variable and independent variables collectively. These data demonstrated homoscedasticity, visually inspected through the scatterplot of the regressed standardised predicted values plotted against the regressed standardised residuals. Values of skewness and kurtsosis were below standard error values and were not collinear, displaying tolerance values between 0.14 and 0.91 (>0.1 indicating non-collinearity). Variance Inflation Factor values were between 1.10 and 7.37 (<10 indicating non-collinearity; Quinn & Keough, 2002; Eisenlohr, 2014).

#### Between year variations

Subsequently, we calculated the difference in the number of calling males between 2015 and 2016, between 2016 and 2017, and between 2015 and 2017. A new binary variable was created to represent encroachment for at least one of three years at each site. We then assessed the difference in the number of calling males between years through Wilcoxon signed tests as the distribution of data was strongly skewed. No assumption was violated as there were no extreme outliers based on box-plots visual inspection, although values for three sites were consistently higher than those for others. Finally, the distribution of paired data set was symmetrical (i.e., match in skewness).

#### Multi-year impact of encroachment

We then assessed the impact of encroachment for the three years on the number of calling individuals and the difference between years. We used an ANCOVA to fulfil the assumptions because the dataset was not normally distributed, but the residuals were. No outliers were detected and studentised residuals were normally distributed under Shapiro–Wilk test of normality (*D* > 0.79, *p* > 0.05). Variances were homogeneous under the Levene’s Test of Equality of Variances (0.97 < *F*_(1,117)_ < 19.43, *p* > 0.05). All covariates were linearly related to the dependent variables, as seen in plot analysis. In the analysis, the six dependent variables were the number of calling individuals for the three years and the difference in the number of calling individuals between years, with encroachment variable as fixed factor and site as covariate. The analysis was conducted under a main effect model for all variables, where statistical significance for the dependent variable is independent of the influence of any other factor.

#### Local extinctions and correction for population-wide variations

Finally, we studied the occurrence of local extirpation through descriptive statistics and spatial visual analysis of heat-maps that are representative of changes in the number of calling males based on interpolation. The interpolation analysis does not include any covariates, such as encroachment, and is solely based on differences in the number of calling individuals. We standardised the difference in the number of calling males at specific localities to detect site-specific deviations from mean population changes. Specifically, for each year-to-year comparison (2015 vs. 2016, 2016 vs. 2017, 2015 vs. 2017) we calculated the difference in the number of calling males between the two years at each site (*x*_*s*_) and the average difference in the number of calling males across all sites for the same two years ($\bar {x}$). We then divided each *x*_*s*_ by $\bar {x}$, yielding ratios that represents site-specific deviations from the overall population differences across all sites. This is important because it cancels range-wide variation, allowing us to detect small scale and site-specific variations. For instance, a cold wave may result in a lower number of calling individuals species-wide, and a dip lower than the established species-wide baseline for the year will highlight a decrease in the number of calling individuals at that site. This analysis allowed us to generate two sets of maps: one of actual change in calling male abundance and one of change corrected for total population change, to show more generalised trends of population change across the species’ range.

Heat maps of actual and corrected change in calling male individuals were created in ArcMap 10.5 Desktop (Environmental Systems Resource Institute, Redlands, CA, USA) using “Local Polynomial Interpolation” across the species’ range. This method was selected as it provides a basic interpolation of changes in calling male abundance with coverage over the entire suitable range. Further, it allows for representation of local variations across the species’ range. We used the default settings (exponential kernel function with one order of polynomial) with a smoothing search neighbourhood. These parameters allowed us to create maps of generalized landscape-scale trends over the study period. All statistical analyses were performed with SPSS v21.0 (SPSS, Inc., Chicago, IL, USA). All geographical inferences were run through ArcMap 10.5 (Environmental Systems Resource Institute, Redlands, CA, USA).

## Results

### Relationship between number of individuals and environmental variables

**Figure 1:**
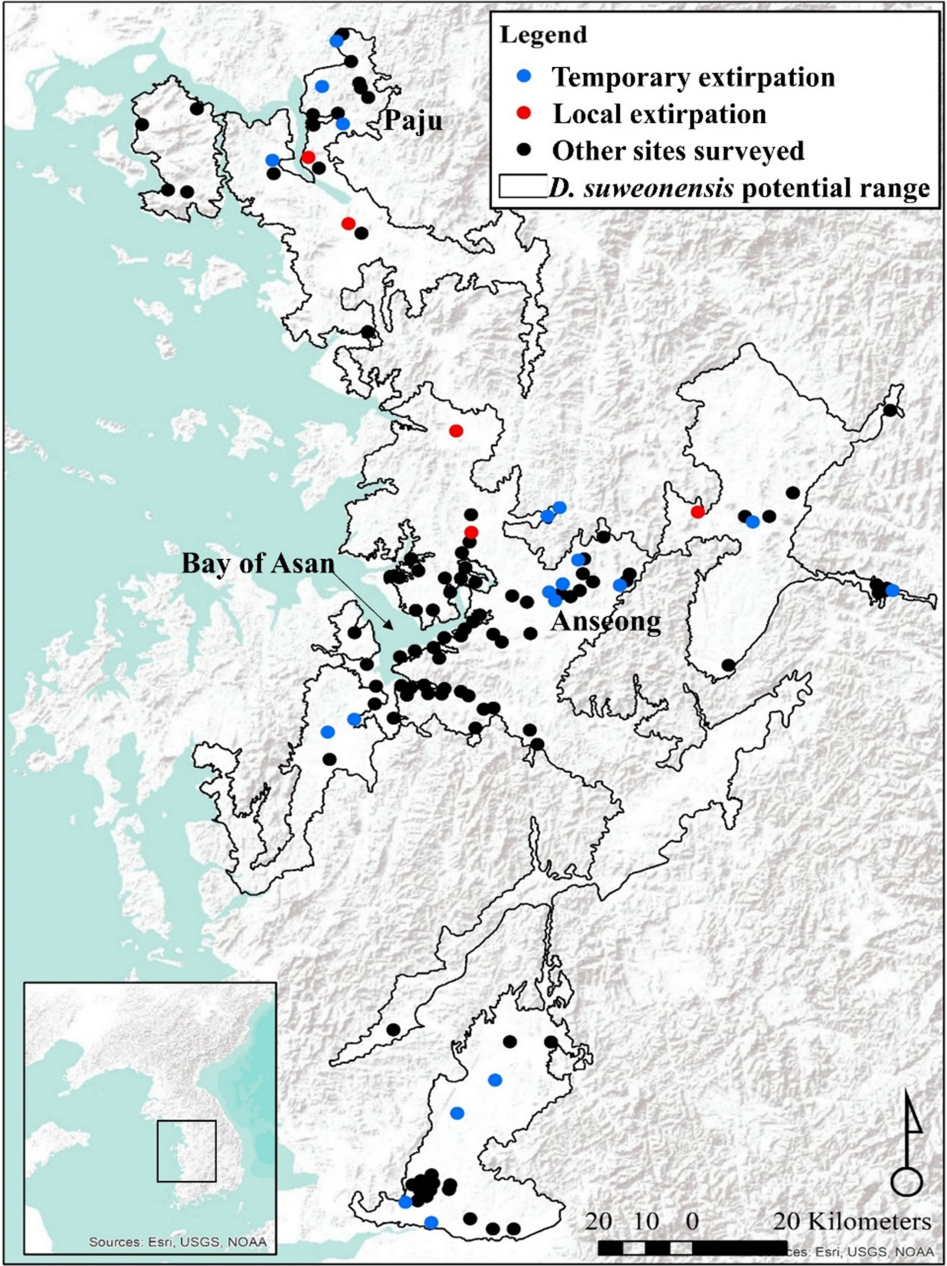
Locations of survey sites with local and temporary extirpations defined over four years. The species range is drawn from Borzée et al. (2017a). Map generated through ArcMap 10.5 (Environmental Systems Resource Institute, Redlands, California, USA), with Service Layer Credits & Sources to Esri, USGS, NOAA and GeoServicesMap Esri Korea.

**Figure 2:**
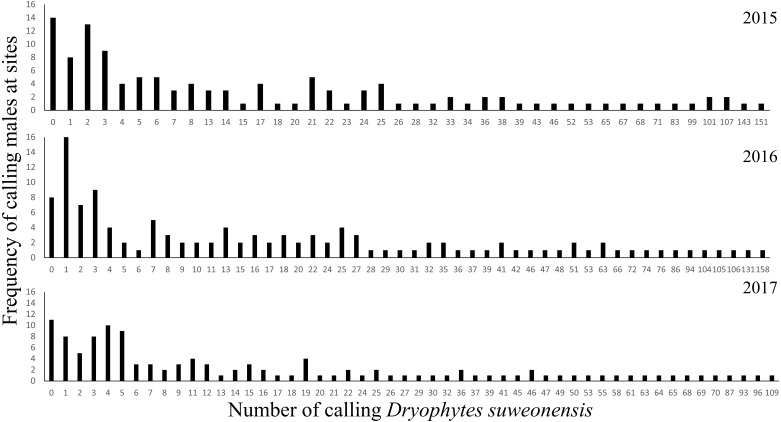
Frequency of the number of calling *D. suweonensis* at the sites surveyed in 2015, 2016, and 2017. The highest number of calling males at a single site was recorded in 2016 while the lowest was recorded in 2017.

Total number of individuals was at an average of 2,510 ± 220.74 over the three years of this study. The number of calling individuals at a site was generally low and linked to several factors. On average, there were 20.74 ± 27.62 (mean ± SD) calling males at a site (*n* = 122 per year), although two sites in 2015 and one site in 2017 were not visited. Thirty-three surveys failed to detect a single calling male (11 sites in 2015, eight sites in 2016, and 14 sites in 2017) for a total of 19 non-overlapping sites (Fig. 1) despite the presence of calling individuals at all sites in 2014. The maximum number of calling individuals at a single site changed between years: it was 109 in 2015, 158 in 2016, and 151 in 2017 (Fig. 2).

The results of the analysis to determine factors important for the number of calling males through the GLMM resulted in a statistically supported model (Omnibus test; *χ*^2^ = 9468.28, *df* = 281, *p* < 0.001) when comparing the fitted model against the intercept-only model. Within the predictive variables, site, date, time of day, water conductivity, CI of *P. chosenicus*, occurrence of *L. catesbeianus*, and encroachment were significant (Table 1). It is interesting to note that the likelihood ratio for encroachment ranked fifth, following site, date, time and CI of *P. chosenicus*.

**Table 1:** Relationship between the number of calling individuals and abiotic and biotic factors. Results obtained through a Generalised Linear Model and with significant variables are highlighted in bold.

	Likelihood ratio *χ*^2^	Error	Coefficient	*df*	*p-*value	Minimum	Maximum	Mean	SD
site ID	1,392.64	1,464.23	12.29	114	**<0.001**				
Date	9,344.14	178.82	2.54	49	**<0.001**				
Time	7,456.60	227.66	1.16	91	**<0.001**				
Wind speed	85.41	0.2	1.2	1	0.662	0	4	1.35	1.27
Cloud cover	10.62	0.65	1.50	1	0.42	0	4	1.66	1.85
Rain intensity	47.28	0.22	0.66	1	0.638	0	1	0.02	0.15
Light intensity	83.90	0.38	1.18	1	0.538	1	498,000	6,971.30	31,642.67
Air pressure	40.92	0.35	0.57	1	0.552	977.70	1,034.00	1,012.48	9.90
Night cover	41.60	0.05	0.58	1	0.943	1	5	3.50	1.29
Moon	7.84	3.33	0.11	1	0.943	0	100	41.14	32.06
Water pH	3.82	0.56	0.05	1	0.455	7.20	10.10	8.39	0.48
Water conductivity	73.42	5.59	1.03	1	**0.018**	4.75	5750.00	903.10	784.68
Air temperature	31.18	2.65	0.52	1	0.104	15.30	36.60	21.87	3.15
Air humidity	6.84	3.05	0.09	1	0.081	23.50	422.00	65.27	25.80
CI *D. japonicus*	0.71	0.77	0.01	1	0.381	0	31	2.42	3.02
CI *P. nigromaculatus*	11.24	0.55	0.15	1	0.460	0	3	1.05	1.09
CI *P. chosenicus*	239.18	22.75	3.38	1	**<0.001**	0	20	1.03	1.42
Occurrence *L. catesbeianus*	187.17	8.07	2.64	1	**0.005**	0	3	0.15	0.49
Encroachment	284.47	12.9	3.51	1	**<0.001**	0	1	0.17	0.38

### Between year variations

The average number of calling individuals was lower for 2015 (19.20 ± 23.68) and 2017 (20.52 ± 29.64) than that for 2016 (22.48 ± 29.21). However, when testing for the difference in the average number of calling *D. suweonensis* between paired years, no significant differences were found in the number of calling males between 2015 and 2016 (Wilcoxon signed tests; *Z* =  − 0.86, *p* = 0.391) or between 2015 and 2017 (*Z* =  − 1.25, *p* = 0.211). Oppositely, a significant difference in the number of calling males was found between 2016 and 2017 (*Z* =  − 2.15, *p* = 0.032).

**Figure 3:**
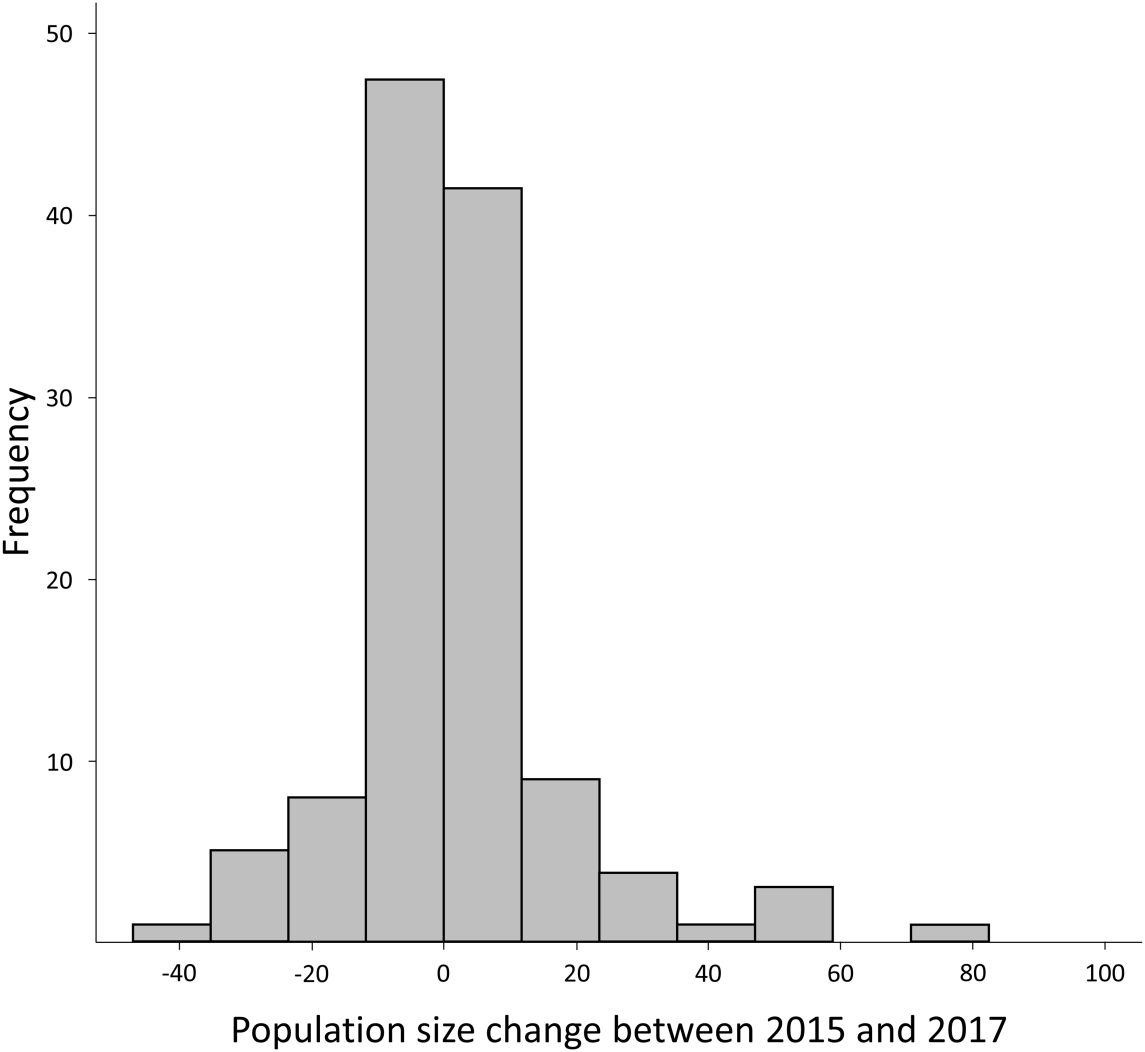
Frequency histogram for the difference in number of calling males between 2015 and 2017. Although negative difference reached −41 individuals and positive variation reached 94 individuals, 90.00% of the variation was within −20 and +20 individuals.

The average for the difference in the number of calling *D. suweonensis* per site between years was positive between 2015 and 2016 (3.60 ± 19.26; mean ± SD), with a maximum drop of 45 individuals (1.64% of 2016 population) and a maximum increase of 112 (4.08% of 2016 population). However, the difference in the average number of calling *D. suweonensis* per site was negative between 2016 and 2017 (−2.13 ± 11.37), with a maximum drop of 74 (2.98% of 2017 population) and a maximum increase of 24 individuals (0.96% of 2017 population). The trend in the average number of calling *D. suweonensis* per site over the three years was positive (2017–2015: 1.47 ± 18.16), with a maximum drop of 41 individuals (1.65% of 2017 population) and a maximum increase of 97 (3.90% of 2017 population). Frequencies for population fluctuation were comparatively higher for minimal differences, with 39.4% of differences for the range [−2:+2] individuals, equal to [−0.07: +0.07]% of the average population size, and with the 10 (= 0.40%) positive and negative extremes cumulated accounting for 11.2% of difference (Fig. 3). Accordingly, the difference in the number of calling individuals between years was significant between 2015–2016 and 2015–2017 (*Z* =  − 2.23, *p* = 0.025), although it was not significant for other periods (2015–2016 and 2016–2017: *Z* =  − 0.09, *p* = 0.925; 2015–2017 and 2016–2017: *Z* =  − 0.66, *p* = 0.508). Such non-significances of some pairs, despite a higher total number of calling individuals in 2016 (Table 2), might be due to lower variations in difference in the number of calling males: 40 for 2015–2017 and 59 for 2015–2016.

**Table 2:** Descriptive statistics for the difference in the number of calling individuals over two pairs of successive years and the 3-year period. The highest number of individuals was recorded in 2016, although a lower range of variations was found between 2016 and 2017.

	Mean	SD	Minimum	Maximum	Range
Difference 2015–2016	3.6	19.06	−45	112	157
Difference 2016–2017	−2.13	11.37	−74	24	98
Difference 2015–2017	1.47	18.17	−41	97	138

While some sites were significantly correlated with the number of calling males in 2015, (*r* = 0.25, *p* = 0.005), there was no significant correlation between sites and the number of calling individuals in 2016 (*r* = 0.11, *p* = 0.239) nor 2017 (*r* = 0.15, *p* = 0.102). It was not significant for any of the three calculated differences in the number of calling males between breeding seasons (0.07 < *r* < 0.14, *p* > 0.131). However, site was correlated with encroachment in 2017 (*r* = 0.18, *p* < 0.045), although it was not correlated with encroachment in other years (2015: *r* = 0.11, *p* = 0.220; 2016: *r* = 0.16, *p* = 0.079). These yearly variations reflected fluctuating dynamics from year to year.

### Multi-year impact of encroachment

Encroachment was found to have a significantly negative impact on the number of calling males. The mean population at sites without encroachment was 24.04 ± 26.87 (*n* = 363). However, it was only 8.80 ± 14.39 for sites with encroachment during the study period (Tables 3 and 4). The same trend was visible for the difference in the number of calling males between years, although the difference was not statistically significant, with the average for sites with encroachment lower than that for sites without encroachment for all three years (Tables 3 and 4). The results of the ANCOVA were supported by a significant model (Wilk’s Lambda; *L* = 0.89, *F*_(4.49,3.00)_ = 114.00, *p* = 0.005), highlighting a significantly negative impact of encroachment on the difference in the number of calling *D. suweonensis* at sites (Tables 3 and 4).

**Table 3:** Descriptive statistics for the impact of encroachment on the number of calling individuals. The data is presented for each of the three years, as well as for the difference in population size between paired-years. Δ is used for “difference between”.

	No-encroachment		Encroachment	
	Mean	SD	Mean	SD
Number of calling males 2015	24.04	26.87	10.57	13.65
Number of calling males 2016	28.61	33.17	10.84	15.55
Number of calling males 2017	27.43	34.27	8.8	14.39
Δ 2015–2016	4.57	21.41	0.27	13.25
Δ 2016–2017	−1.19	9.96	−2.05	8.42
Δ 2015–2017	3.39	20.8	−1.77	10.86

**Table 4:** Results of the ANCOVA for the variations in the number of calling individuals. It includes the number of calling individuals in 2015, 2016 and 2017, as well as the difference in the number of calling individuals between years (dependent variable), encroachment (fixed factor) and site ID (covariate). Significant variables are highlighted in bold. Δ is used for “difference between”.

		Mean square	*df*	*F*	*p*-value
Encroachment	2015	3,765.31	1	7.48	0.007
	2016	7,952.69	1	10.09	0.002
	2017	8,377.91	1	10.27	0.002
	Δ 2015–2016	773.71	1	2.23	0.138
	Δ 2016–2017	5.54	1	0.06	0.802
	Δ 2015–2017	910.15	1	2.88	0.092
Site ID	2015	3,042.16	1	6.04	0.015
	2016	423.1	1	0.54	0.465
	2017	1,154.2	1	1.41	0.237
	Δ 2015–2016	1,196.21	1	3.44	0.066
	Δ 2016–2017	179.67	1	2.04	0.156
	Δ 2015–2017	448.69	1	1.42	0.236
Error	2015	503.27	116		
	2016	788.07	116		
	2017	816.1	116		
	Δ 2015–2016	347.3	116		
	Δ 2016–2017	88.05	116		
	Δ 2015–2017	315.99	116		

### Local extinctions and correction for population-wide variations

Compared to confirmed occurrence in 2014, populations went locally extirpated, although sometimes temporarily, at 13 sites in 2015, eight sites in 2016, and 15 sites in 2017. A total of five sites were devoid of calling *D. suweonensis* for three years in a row, while calling males were not found at two sites for two years. Out of 21 sites where extirpation occurred, only six recovered their populations (Fig. 1).

When correcting for the general average difference in the number of calling males, the average standardised difference in population size at sites was positive for 2015–2016 (3.78 ± 19.15 individuals = 0.15 ± 0.76% of averaged population sizes), but negative for 2016–2017 (−2.06 ± 11.41 individuals = 0.08 ± 0.43% of averaged population sizes) and 2015–2017 (−16.59 ± 27.39 individuals = 0.69 ± 1.14% of averaged population sizes). This highlights a general negative trend in population size between 2015 and 2017, despite a non-significant range-wide increase trend on the same period, which was driven by a few sites with large population gains. This is important as non-corrected trends are necessary to describe large scale variations in the number of calling individuals, and thus perfectly illustrate the impact of ecological variables such as climate change, but they fail at describing local variations in relation with the general pattern of population trends. For instance, a dry year will result in a decrease in the number of calling individuals population-wide, but a dip in the number of calling individuals at a site lower than the population-wide average of that year will demonstrate the importance of local factors.

**Figure 4:**
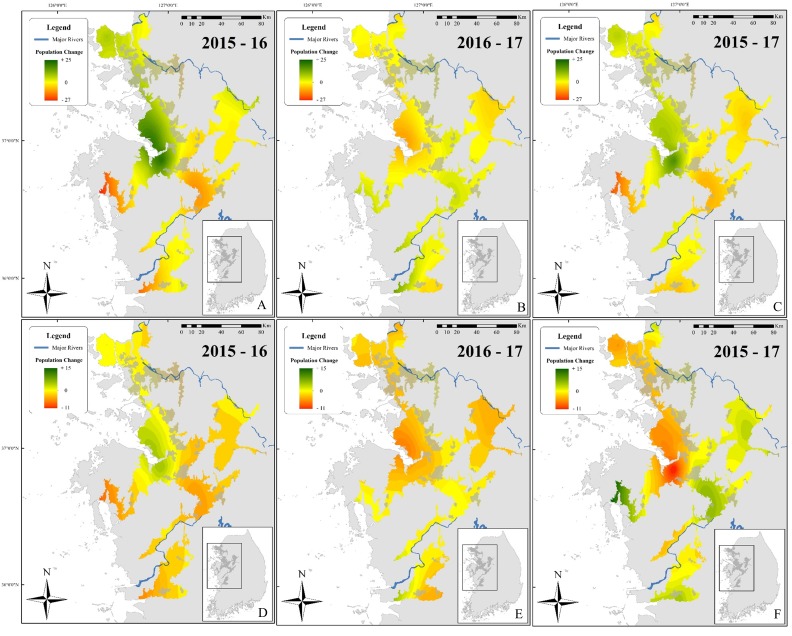
Maps showing differences in the number of calling *D. suweonensis* between 2015 and 2017. The species range is drawn from Borzée et al. (2017a) and Borzée et al. (2017b). Maps showing variation in the number of calling males between (A) 2015 and 2016, (B) 2016 and 2017, and (C) 2015 and 2017. Maps D, E, and F followed the same time series as A, B, and C, respectively, although they were based on corrected differences in the number of calling individuals to reflect differences at sites, independently of variations in the whole population. Maps were based on Kriging interpolations and generated through ArcMap 10.5 (Environmental Systems Resource Institute, Redlands, California, USA), with Service Layer Credits to DIVA-GIS (for administrative boundaries). Red represents a decrease in the number of calling individuals while green indicates an increase. Note the unbalanced colour scale.

This spatial analysis (Fig. 4) was based on the potential range of the species after removal of urban areas (see Borzée et al., 2017a). The species was not present in large populations in some parts of the range (see Fig. 1 for populations). Caution should be taken when reading Fig. 4 as the colour gradient is skewed toward a decrease in the number of individuals (dark red), as there were no sites with a sharp increase in number of calling males (green). The non-corrected difference in the number of calling individuals (A, B, and C) was geographically consistent with differences between the two breeding seasons for two localities: on the northern edge of the range (Paju) and at the centre of the range around the Bay of Asan (Fig. 4). For maps D, E, and F, values were standardised using a ratio to remove trends shared across the entire range of the species and highlight variations at sites. This subsequent analysis also supported the decrease in the number of calling individuals in Paju and around the Bay of Asan, although with some variations in intensity and wideness of patterns. The patterns in population variations matched with non-corrected data for respective year-by-year analyses. Interestingly, patterns for 2015–2017 were almost opposite between corrected and non-corrected data, with the exception of the population decline in Paju and Anseong. It should be noted that the difference in the number of calling individuals did not drastically increase anywhere within the range, while it did go down in large number in some areas.

Although these interpolation maps do not represent fine-scale changes based on environmental variables and microhabitats, they are helpful in representing the population trend of *D. suweonensis* during the study period and can help identify geographic regions of the most extreme population decline. Whereas a site-based approach can pinpoint fine-scale decline, interpolation of these trends over the species’ entire range can help identify larger regions of population decline and provide insight into population-scale dynamics over time. Besides, the interpolation analysis does not include any covariates, such as encroachment, and is solely based on differences in the number of calling individuals.

## Discussion

We hypothesised that there would be minimal differences in the number of calling males between seasons due to the relative consistency of agricultural and irrigation practices. This prediction was demonstrated to be relatively correct, as most differences in the number of calling individuals between years were between 2 and −2 individuals (−0.07: +0.07% of averaged population size) per site per season. However, the prediction was wrong in the presence of additional stress factors, such as encroachment, as the population dropped for sites where encroachment was detected. Some populations even went locally extirpated at some sites for non-defined reasons. Overall, we found that the number of calling males, as a proxy for population size, was slowly declining. This trend is likely linked to the destruction of breeding sites through encroachment. Extirpation seemed to be definitive at five sites where no individual was detected over the three years of surveys, despite the species being detected in 2014. The loss of these specific sites will lead to the loss of connectivity between populations south and north of Seoul. In the long term, it will result in a loss of genetic exchange, leading to loss of fitness as already detected in the species for population with low genetic diversity (Borzée, Yu & Jang, in press), and potential extinction (Soulé, 1987; Dodd Jr, 2010).

Furthermore, 33 surveys failed to detect a single calling male at a total of 19 sites, representing 6.42% of sites where the species was known to occur. Indeed, large variations in population sizes can be expected between years (Semlitsch et al., 1996; Taylor, Scott & Gibbons, 2006; Bevelhimer et al., 2008; McCain, Szewczyk & Bracy Knight, 2016), although they usually do not lead to the total absence of individuals at sites. Despite a generally dryer climate in 2016, this absence of individuals is meaningful as large and healthy populations of Hylids are usually characterised by a detection probability >0.9 (Petitot et al., 2014). Although *D. suweonensis* was present at some of these sites in the subsequent year, some populations are at risk of becoming locally extirpated (Blaustein, Wake & Sousa, 1994) and potentially functionally extinct.

The corrected trend over the three years highlights a slow decline in the total number of *D. suweonensis* individuals (Fig. 4F) and is estimated to be robust and valid. This brings focus to risks such as the reduction in the surface area of rice-paddy complexes. Even if it only is “a corner” of the site that is cut away, the population is likely to decrease as a result. This threat is exacerbated by the fact that rice paddies are the only remaining breeding habitats of *D. suweonensis* (Borzée & Jang, 2015).

When taking into account the corrected number of calling individuals for range wide trends, the number of calling individuals between 2015 and 2017 decreased. However, this number was a general trend, significant for some pair-years only, and was most likely related to discrete environmental variables (Blaustein & Wake, 1995). When the corrected difference in the number of calling males was compared to the difference in number of individuals on average, we detected a decline in population size at most north-western sites. These sites are in the areas with the largest human populations and are victims of encroachment. Besides, the interpolation analysis may also be subjected to spatial-autocorrelation. For instance, the region including the north-western sites is under government subsidised development, while other regions are not under such an important development pressure. As a result, sites that are spatially related in the north-west will be more likely to see drops in the number of calling individuals in comparison to sites that are not directly contiguous. When looking at population sizes, the general trend was also contradicted by differences in the number of individuals between years as no significant differences were found in the number of calling males between 2015 and 2016 and between 2015 and 2017. These variations may arise from the fact that the interpolation analysis does not include the covariates that are used in the analysis with the non-corrected number of individuals.

Finally, the patterns between corrected map (Fig. 4C) and non-corrected map (Fig. 4F) for the difference in the number of calling males between 2015 and 2017 displayed large differences. This is due to outlier sites with comparatively exaggerated changes in population sizes. This result highlights that, despite a seemingly general increase in population, local patterns can be hidden, and those provide clues for the slow decline in population size. These differences in the number of calling males are discrete in locality year by year, and insidious, as they are invisible until there is a large drop in population size at a site. This is typically difficult to detect as aging males are still detected during call surveys, although not actively breeding due to the reduction in mate availability. Therefore, once a population decline is detected the impact of conservation plans will be weak. These discrete minor differences in the number of calling males are thus strongly negatively impacting the whole dynamics of the species in the long term, and preventive conservation actions are required.

The variation in the number of calling males at sites changed along with environmental variations (e.g., Salvador & Carrascal, 1990; Fukuyama & Kusano, 1992; Oseen & Wassersug, 2002; Llusia et al., 2013) and with the occurrence of the invasive and chytrid fungus spreading-agent *L. catesbeianus* (Borzée et al., 2017b). However, some variables were not significant although expected to be. These were for instance wind speed (Oseen & Wassersug, 2002; Weir et al., 2005), rain intensity (Oseen & Wassersug, 2002; Steelman & Dorcas, 2010), light intensity (Blair, 1961; Navas, 1996; Taylor, Buchanan & Doherty, 2007), water conductivity and CI of *P. chosenicus* (Llusia et al., 2013). Thus, the estimated population size could be modelled from survey data, once corrected for environmental variations. This can be done through the probability of detecting the species based on its ecological preferences (see for instance Guimarães, Doherty & Munguía-Steyer, 2014), although the influence of environmental factors can differ between populations (Berg, Brumfield & Apanius, 2006; Llusia et al., 2013).

## Conclusion

We determined population trends for the endangered Suweon Treefrog (*D. suweonensis*) through aural surveys in 2015, 2016, and 2017 at most of 122 sites where the species was known to occur in the Republic of Korea. We found that the number of calling males was generally declining, with some local extirpations, and that it was influenced by environmental and landscape variables. Encroachment was a key factor that influenced both the number of calling individuals and the difference in the number of calling individuals between years. While there was a large fluctuation in the number of individuals between years at some sites, most sites displayed minimal differences between years. Finally, when correcting for the overall population size variations between given years, but omitting covariates, we detected a decrease in the number of calling individuals over the three years of this study. We emphasize that because of the potential for calling males in remnant populations with low population recruitment, rice paddies have to be preserved before the detection of population decline as they are the only breeding habitat of *D. suweonensis* currently.

##  Supplemental Information

10.7717/peerj.5568/supp-1 Data S1 Dataset on field surveys Raw data for surveys conducted between 2014 and 2017 for Dryophytes suweonensis.

## Additional Information and Declarations

### Data Availability

The following information was supplied regarding data availability: The raw data are included as Data S1.
